# The Role of Chatbot GPT Technology in Undergraduate Dental Education

**DOI:** 10.7759/cureus.54193

**Published:** 2024-02-14

**Authors:** Vinayak A Thorat, Prajakta Rao, Nilesh Joshi, Prakash Talreja, Anupa Shetty

**Affiliations:** 1 Department of Periodontology, Bharati Vidyapeeth (Deemed to Be University) Dental College and Hospital, Navi Mumbai, IND

**Keywords:** dental college, undergraduate, artificial intelligence, simulation in dental education, evidence based learning, dentistry, dental education, chatbot gpt

## Abstract

This comprehensive article explores the transformative role of Chatbot GPT, based on the GPT-3 architecture, in revolutionizing dental education. The focus is on its impact across various facets, including personalized learning pathways, integration into virtual patient simulation scenarios, 24/7 accessibility, multilingual support, interactive dental dictionary functionality, evidence-based learning, and assessment and evaluation of dental students. The objective is to showcase how Chatbot GPT enhances educational experiences, promotes inclusivity, and aligns with contemporary pedagogical principles. Chatbot GPT emerges as a powerful ally in dental education, offering personalized learning experiences, risk-free clinical simulations, continuous accessibility, multilingual support, instant terminology assistance, evidence-based learning resources, and real-time assessment capabilities. Its adaptability caters to diverse learning needs, fostering a learner-centered approach and promoting lifelong learning for both dental students and practitioners. As a versatile tool, Chatbot GPT not only transforms the educational journey but also serves as a valuable asset for continuous professional development in the dynamic landscape of dentistry.

## Introduction and background

Dental education plays a pivotal role in shaping competent and skilled dental professionals. As technology continues to advance, the incorporation of innovative tools in educational settings has become increasingly significant [1,2]. Chatbot GPT, based on the GPT-3 architecture, represents a powerful artificial intelligence (AI) language model capable of understanding, processing, and generating human-like text responses. This transformative technology opens new possibilities for optimizing dental education and empowering students with personalized learning experiences [3].

Traditionally, dental education has heavily relied on didactic lectures, textbooks, and hands-on clinical experiences. While these methods remain fundamental, incorporating technology-driven approaches can augment the learning process and address diverse educational needs [4]. Chatbot GPT's versatility and adaptability empower it to cater to various aspects of dental education. It can provide real-time assistance, offer personalized learning resources, and engage students in interactive scenarios, fostering a more dynamic and tailored educational experience. This adaptability allows it to seamlessly integrate with traditional methods, creating a comprehensive support system for dental students from the beginning to the end of their educational journey [5].

## Review

Review methodology

Literature Search

A systematic search was conducted across electronic databases (Google, Medline, and Education Source) to identify relevant articles published from 2014 to 2023. References from books and websites were also considered to gain deeper insight into the topic. Keywords included “Chatbot GPT,” “undergraduate dental education and AI,” “Artificial intelligence in dentistry,” and various related terms and phrases.

Inclusion/Exclusion Criteria

Articles, reviews, conference papers, and notes were included if they focused on the integration of GPT-based chatbots in undergraduate dental education, provided empirical evidence, and were published in English. Exclusion criteria comprised studies outside the specified time frame or those not directly related to dental education. The language was restricted to English.

Data Extraction

Key information, such as study design, GPT technology implementation details, and educational outcomes, was systematically extracted from the selected articles. This facilitated a comprehensive analysis of the impact and effectiveness of Chatbot GPT technology in dental education. All the coauthors equally contributed to collecting the articles. Information of relevant importance was included in the review article.

Personalized learning pathways

Personalized learning pathways offered by Chatbot GPT have the potential to revolutionize dental education by providing individualized support to students based on their specific needs and learning styles. The AI language model's ability to generate context-specific responses allows it to comprehend students' queries and deliver personalized recommendations. By understanding students' strengths and weaknesses through previous interactions, the chatbot can adapt its responses and provide tailored study materials, resources, and practice exercises that cater to their unique learning preferences.

Research in educational technology and AI-driven personalized learning has demonstrated its efficacy in improving students' learning outcomes and engagement. A study by Yovanoff et al. explored the impact of personalized learning systems in the context of medical education and found that individualized learning paths significantly enhanced students' performance and knowledge retention [6]. Similarly, a study by Maier and Klotz investigated personalized learning algorithms in higher education and reported improved student satisfaction and learning outcomes [7].

The use of AI-driven personalized learning systems has gained traction in various educational fields, including healthcare and medicine. Dental education can benefit from such technology by empowering students to take control of their learning experience and focus on areas where they need additional support.

Providing students with tailored study materials and resources helps create a dynamic and adaptive learning environment, enhancing students' motivation and self-directed learning.

Furthermore, personalized learning pathways through Chatbot GPT align with the principles of student-centered learning, a pedagogical approach that prioritizes students' needs, interests, and individual progress [8].

A detailed explanation is given next.

Individualized Content Delivery

Chatbot GPT can analyze individual student preferences, learning styles, and performance data to deliver content tailored to each student's specific needs and interests. By understanding the pace at which a student learns best, the chatbot can adapt the complexity and format of content, ensuring it matches the student's progress.

Autonomous Learning Opportunities

Student-centered learning emphasizes enabling students to learn at their own pace. Chatbot GPT can offer self-paced learning experiences, enabling students to delve deeper into topics of interest or revisit challenging concepts without feeling rushed. Students have the autonomy to choose learning pathways and topics aligned with their interests, fostering a sense of ownership and motivation in their educational journey.

Tailored Feedback and Support

Chatbot GPT can provide immediate and personalized feedback, addressing specific strengths and weaknesses in a student's understanding of dental concepts. Recognizing individual challenges, the chatbot can offer additional resources, explanations, or alternative learning approaches, aligning with the principle of addressing individual needs.

Engagement and Motivation

Creating learning scenarios relevant to dental practice enhances engagement. Chatbot GPT can simulate real-world cases, making the learning experience more meaningful and motivating for students pursuing a career in dentistry. Integration of gamification elements, such as quizzes or interactive scenarios, adds an element of fun and competition, further aligning with the principles of student-centered learning that value engagement.

Continuous Assessment and Progress Tracking

Chatbot GPT can monitor students' progress in real-time, allowing educators and students to track their development and address challenges promptly. Based on continuous assessment, the chatbot can dynamically adjust learning pathways, ensuring that students receive content and challenges that match their evolving skill levels.

Collaborative Learning Opportunities

Student-centered learning encourages collaboration. Chatbot GPT can facilitate peer interactions, discussions, or collaborative projects, promoting a community of learners while leveraging the technology for meaningful engagement.

The following are some limitations to personalized learning:

First, while AI endeavors to tailor learning experiences to individual students, it often grapples with the precision required to truly understand each student’s unique needs and preferences. Despite its sophisticated algorithms, the variability in learning styles and preferences presents a significant challenge, potentially limiting the effectiveness of personalized content delivery. Moreover, the emotional intelligence necessary for effective personalized education is often beyond the current capabilities of AI systems. Understanding and responding to the emotional state of a student, particularly in a complex field like dentistry, poses a considerable challenge that warrants further exploration.

Data privacy concerns loom large in the realm of personalized education. The collection and analysis of vast amounts of student data raise ethical dilemmas surrounding privacy and data security, especially in healthcare-related fields like dentistry, where sensitive information is involved. Safeguarding the privacy of students’ data while harnessing the power of AI remains an ongoing challenge. Furthermore, the adaptability of AI algorithms to diverse learning styles is not without its limitations. While some students may thrive in hands-on experiences, others may prefer theoretical approaches. Crafting a truly adaptable AI system capable of accommodating these variations presents a significant challenge in the field of personalized education.

Additionally, the efficacy of AI in personalized dental education hinges heavily on the quality and diversity of input data. Biases in data or a limited dataset could skew recommendations, hindering the system’s ability to provide well-rounded and unbiased educational content. While AI can assist in theoretical aspects of dental education, the practical, tactile skills essential in dentistry may not be effectively addressed through current technologies. Bridging this gap between theory and practice remains a formidable challenge in the integration of AI into dental education.

Ethical considerations loom large in the implementation of personalized education through AI. Ensuring that AI-driven educational systems are ethically sound, and unbiased, and promote diversity and inclusivity is a complex task that requires careful consideration and ongoing vigilance.

Finally, the high initial implementation costs associated with integrating AI into dental education may pose a barrier to accessibility, particularly in resource-constrained educational settings. Balancing the potential benefits of personalized education with the financial investment required is a challenge that educators and institutions must grapple with as they navigate the evolving landscape of dental education. Despite these limitations, ongoing advancements in AI and continuous efforts to address these challenges hold the potential to enhance personalized dental education and improve the learning experiences of students in the field.

AI platforms available in the public domain can be helpful in student education, each with its advantages and disadvantages.

​​​OpenAI's GPT-3 stands out for its versatility, adeptness in understanding natural language, and ability to execute a wide spectrum of tasks. However, its reliance on API access and limitations in response length necessitates careful management. Google’s BERT excels in natural language understanding and context-aware processing, but its resource-intensive nature and computational demands may pose challenges. Microsoft Azure Cognitive Services offers a comprehensive suite of AI services, from computer vision to language understanding, yet extensive usage can incur significant costs and certain domain limitations. IBM Watson presents a robust platform with services spanning natural language processing and machine learning, though its complexity may present a steep learning curve for some users. Facebook AI provides valuable tools and libraries, particularly PyTorch for deep learning tasks, yet its ecosystem limitations may restrict its applicability compared to more general-purpose models. TensorFlow, a product of Google, boasts widespread adoption for machine learning and deep learning, but its steep learning curve may deter beginners. PyTorch is esteemed for its dynamic computational graph and user-friendly interface, though historically perceived as less production-friendly than TensorFlow. Microsoft’s Bing Co-Pilot offers code suggestions for programming tasks, which can be invaluable, but vigilance is necessary to ensure code quality, and coverage across programming languages may vary.

Integrating chatbot GPT into virtual patient simulation scenarios

Chatbot GPT can enhance virtual patient simulations for undergraduate dental students by serving as a virtual assistant. It could provide realistic patient interactions, simulate diverse scenarios, and offer instant feedback on diagnostic and treatment decisions. Integrating GPT into these simulations can help students practice communication skills, refine clinical reasoning, and receive personalized guidance, contributing to a more immersive and effective learning experience.

Dental colleges can implement AI infrastructure in various ways to enhance the integration of Chatbot GPT into virtual patient simulation scenarios education within educational settings.

Dental colleges can develop AI-powered virtual patients that mimic real-world dental cases, allowing students to practice diagnosis and treatment planning in a risk-free environment. They can utilize AI for image recognition and analysis in radiology, aiding in the diagnosis of dental conditions through automated analysis of X-rays and scans. Implementing AI-driven adaptive learning systems that tailor educational content to individual students' needs ensures a more personalized and effective learning experience. By integrating AI-powered chatbots, colleges can assist students with questions, provide instant feedback on coursework, and offer additional resources for further understanding.

Colleges can also develop AI systems to assist dental practitioners in making informed decisions during patient care, considering factors like patient history and current clinical data. By using AI to analyze large datasets for dental research, colleges can identify patterns, trends, and potential breakthroughs in oral health. Implementing AI-driven telehealth solutions enables remote consultations, enhances access to expert advice, and promotes continuous learning beyond the traditional classroom setting. Finally, colleges can explore the use of AI-driven robotics for certain dental procedures, supporting precision and efficiency in tasks like surgeries or repetitive procedures.

Research in medical and dental education has shown the benefits of virtual patient simulation in enhancing students' clinical competence and critical thinking abilities. The findings of a systematic review by Rania et al. suggested making affordable VR hardware and software easily accessible for the development of secure and cost-effective interactive educational training. This would enable learners and trainees to engage instantly through their personal computers or mobile devices. It is recommended to clearly define learning content and determine the extent to which conventional workflows should be included alongside virtual content [9]. Similarly, a study by Li et al. explored the use of virtual patient simulation software in dental education, demonstrating its effectiveness in improving students' clinical performance and confidence [10].

By immersing students in realistic clinical scenarios, virtual patient simulations allow them to apply theoretical knowledge to practical situations, bridging the gap between classroom learning and real-world clinical practice. Chatbot GPT's ability to generate context-specific responses further enhances the simulation experience, as students can interact with the AI model as if it were a virtual patient, receiving feedback and guidance on their decisions.

Moreover, virtual patient simulation with Chatbot GPT enables students to explore a wide range of dental cases, including rare and complex dental scenarios, including simulating rare conditions, multidisciplinary cases, challenging diagnoses, emergencies, and patient communication challenges. This exposure helps students refine their skills and readiness for diverse challenges they may encounter in their future dental practice. This exposure to diverse cases cultivates a well-rounded clinical experience and prepares students for real-life challenges they may encounter in their dental careers.

The use of virtual patient simulation aligns with the principles of experiential learning, where students actively engage in hands-on experiences to develop skills and knowledge [11]. By repeatedly practicing in a risk-free environment, students can refine their clinical decision-making processes and learn from mistakes without adverse consequences.

The 24/7 accessibility of Chatbot GPT in dental education

The 24/7 accessibility of Chatbot GPT in dental education is a game-changer, empowering students to access educational support and guidance whenever they need it, even outside of traditional classroom hours. This AI-driven tool operates seamlessly around the clock, ensuring continuous availability to students, irrespective of their time zone or schedule. With Chatbot GPT, students have the flexibility to access educational content, seek assistance, and engage in learning activities at their convenience, promoting self-directed and independent learning.

Studies on the benefits of technology-driven accessibility in education have shown positive impacts on students' learning outcomes and motivation. A study by Goundar and Kumar explored the use of mobile learning applications and found that 24/7 accessibility led to increased engagement and learning efficiency among students [12].

Students can revisit previously covered dental topics at their convenience. This helps in reinforcing knowledge and improving retention, as repetition is essential for learning. The chatbot allows students to seek clarification on any uncertainties they may have. This instant clarification ensures that misunderstandings are addressed promptly, preventing them from becoming obstacles to further learning. Through interactive conversations, the chatbot helps reinforce students' understanding of dental concepts. This dynamic engagement aids in solidifying knowledge and applying it in practical scenarios.

The key advantage lies in the on-demand nature of the support. Students can engage with the chatbot at any time, accommodating different schedules and preferences. This is particularly beneficial for those who may have irregular study patterns or time constraints. Recognizing that students learn at different speeds, the chatbot's continuous support caters to individual learning paces. Some students may grasp concepts quickly, while others may require more time. The chatbot adapts to these variations, ensuring a personalized learning experience.

The on-demand accessibility empowers each student to progress through the material at their speed. This autonomy is valuable as it accommodates diverse learning preferences, contributing to a more effective and enjoyable learning journey.

The promotion of self-directed learning through Chatbot GPT aligns with the principles of learner-centered pedagogy, where students take an active role in their learning process [13]. By having constant access to educational resources and assistance, students can take ownership of their learning journey and develop a sense of responsibility for their academic progress [14].

Multilingual support offered by Chatbot GPT in dental education

The multilingual support offered by Chatbot GPT in dental education is a significant advancement, as it addresses the diverse linguistic needs of students from various cultural backgrounds. This AI language model's ability to comprehend and respond in multiple languages enables it to cater to a wide range of student populations, breaking down language barriers and promoting inclusivity in dental education.

Studies on the importance of multilingual support in educational technology have shown its positive impact on students' learning experiences and engagement. A study by Athanassopoulos et al. examined the use of multilingual chatbots in language learning and reported improved language proficiency and communication skills among students [15]. Similarly, a study by Mehdi et al. explored the benefits of multilingual education tools, emphasizing the role of technology in fostering a culturally diverse and inclusive learning environment [16]. 

By accommodating multiple languages, Chatbot GPT ensures that students who are more comfortable in their native language can actively participate in the learning process. This inclusivity fosters a sense of belonging and equity, making dental education accessible to students from diverse linguistic backgrounds.

Moreover, multilingual support in Chatbot GPT promotes cross-cultural communication and collaboration among students, enriching the learning experience with diverse perspectives and experiences. Dental students from different countries and regions can interact, exchange knowledge, and learn from each other, contributing to a more globally aware and interconnected dental community.

The integration of multilingual support aligns with the principles of culturally responsive teaching, where educators acknowledge and respect students' cultural identities and language backgrounds [17]. By providing content and support in students' preferred languages, Chatbot GPT creates a learning environment that is sensitive to individual cultural and linguistic needs.

Chatbot GPT's role as an interactive dental dictionary

Chatbot GPT's role as an interactive dental dictionary is invaluable in supporting dental education by offering instant access to a wide range of dental terminology and definitions. This AI language model can efficiently provide students with clear and concise explanations of dental terms, aiding them in building a robust dental vocabulary. As students encounter unfamiliar or complex terms during their studies, they can simply interact with Chatbot GPT to obtain quick and accurate definitions, enhancing their understanding of various dental concepts.

While both ChatGPT and Google Search involve language processing, ChatGPT is conversational and generates text dynamically based on input, making it suitable for interactive tasks. Google Search, on the other hand, focuses on retrieving information from the internet in real time based on keyword queries, providing links to relevant web pages. They serve different purposes, with ChatGPT being more interactive and Google Search being a powerful tool for information retrieval.

Research on the use of interactive language models in education has demonstrated their efficacy in promoting vocabulary development and language comprehension. A study by Trentin explored the use of interactive language models in language learning settings and found that students who interacted with the AI model improved their vocabulary knowledge and retention [18]. ChatGPT aids in knowledge and vocabulary retention through interactive conversations, contextual learning, quizzing, instant clarification, personalized responses, language practice, and exploration of synonyms. Its adaptability and 24/7 accessibility offer users a dynamic tool for reinforcing learning and language skills. Similarly, a study by Hsu et al. investigated the benefits of using AI-based interactive tools for medical terminology learning and reported improved learning outcomes and knowledge acquisition among medical students [19].

The interactive dental dictionary feature of Chatbot GPT offers students a user-friendly and efficient way to access dental terminology. This aspect not only aids students in understanding essential dental concepts but also helps them communicate effectively with peers and dental professionals. By consistently engaging with Chatbot GPT for terminology exploration, students can develop a strong dental vocabulary, which is crucial for their success in academic and clinical settings.

Chatbot GPT and evidence-based learning

Chatbot GPT's provision of evidence-based learning resources is a valuable asset in dental education, empowering students to access a vast repository of dental research papers and evidence-based literature. By having access to a comprehensive collection of scholarly articles and studies, students can make informed decisions based on the best available evidence in their clinical practice and academic pursuits. This AI-driven tool fosters critical thinking and research skills among dental students, encouraging them to critically analyze and apply evidence to make sound clinical judgments.

Studies on the impact of evidence-based learning tools in medical and healthcare education highlight their role in promoting evidence-based practice and research skills. A study by Zeri et al. examined the effects of evidence-based learning interventions in healthcare settings and reported significant improvements in students' knowledge, skills, and attitudes towards evidence-based practice [20]. Similarly, a study by Shehzad et al. investigated the use of evidence-based learning in medical education and found that students exposed to evidence-based resources exhibited enhanced research skills and information literacy [21].

Chatbot GPT's provision of evidence-based learning resources empowers dental students to stay updated with the latest advancements and research findings in the dental field. By critically evaluating and integrating evidence into their clinical decision-making, students can provide high-quality and patient-centered dental care.

Chatbot GPT's role in the assessment and evaluation of dental students

Undergraduate dental curricula heavily rely on knowledge-based assessments, serving as a fundamental element to showcase that dental graduates possess the essential scientific knowledge for informed clinical practice. Traditionally, these assessments were conducted face-to-face in university settings, utilizing pen and paper. A study conducted by Ali et al. marked a groundbreaking exploration into the influence of generative AI, exemplified by ChatGPT, on conventional assessments within dental education. The results showcased ChatGPT's proficiency in tackling dental assessments, yielding satisfactory grades. These findings align with recent studies indicating ChatGPT's ability to perform on par with or near the passing threshold across all three sections of the United States Medical Licensing Examination® without requiring additional training or reinforcement [22].

The outcomes emphasized the necessity for dental education providers to acknowledge the impact of rapid technological advancements on dental education. They underscore the importance of devising strategies to counteract inappropriate technology usage while ensuring that both students and faculty can effectively harness the benefits of this technology. This challenge is not unprecedented, as education providers have previously grappled with the transformative impact of innovative technologies. The current generation of academics has witnessed the internet revolution, reshaping information access through powerful search engines like Google, web-based applications such as YouTube, and the adoption of digital flashcards. Unlike the pre-millennium era, teachers and textbooks no longer serve as the exclusive sources of information for students.

The use of Chatbot GPT for formative assessments fosters a learner-centered approach, where students take an active role in their learning process and receive personalized feedback tailored to their responses. The chatbot's AI capabilities enable it to adapt to individual learning needs, providing targeted explanations or additional resources based on students' quiz performance [23].

Moreover, the real-time nature of the assessments offers immediate insights to instructors on students' understanding of specific topics or concepts. Instructors can analyze students' responses and identify common misconceptions or areas that need further emphasis in the curriculum. This data-driven approach allows educators to tailor their teaching strategies and provide targeted support to individual students.

By incorporating assessment opportunities throughout the learning journey, students are encouraged to engage actively, reflect on their learning progress, and strive for continuous improvement [24].

During the pandemic, dental institutions undertook a widespread transition to remote online assessments, with some utilizing commercially available platforms designed for appropriate student proctoring. However, many institutions, especially in developing countries, relied on open-source online platforms such as Zoom and Webex. While these platforms allowed observation via camera during assessments, they didn't offer the ability to restrict candidates' internet access. To address this, some institutions created *nonsearchable* questions to hinder quick internet searches. However, the emergence of bots like ChatGPT raises concerns about the appropriateness of this approach for remote assessments conducted without proctoring [25]. 

## Conclusions

In conclusion, the integration of Chatbot GPT in dental education offers promising avenues for transforming the learning experience. By providing personalized learning pathways aligned with student-centered principles, Chatbot GPT fosters adaptive environments tailored to individual needs. Despite the benefits, it's important to acknowledge limitations such as precision challenges and ethical concerns. However, the use of Chatbot GPT in virtual patient simulations enriches practical training, while its accessibility promotes flexible learning. Multilingual support fosters inclusivity and its role as an interactive dictionary aids in language development. Moreover, its provision of evidence-based resources enhances critical thinking. Moving forward, collaborative efforts are needed to strike a balance between embracing innovation and addressing challenges, ensuring the continual evolution of dental education to meet the needs of students and the profession.
